# Modulating hemispheric lateralization by brain stimulation yields gain in mental and physical activity

**DOI:** 10.1038/s41598-017-13795-1

**Published:** 2017-10-18

**Authors:** Catharina Zich, Siobhán Harty, Cornelia Kranczioch, Karen L. Mansfield, Francesco Sella, Stefan Debener, Roi Cohen Kadosh

**Affiliations:** 1 Department of Psychology, University of Oldenburg, 26111 Oldenburg, Germany; 2 Department of Experimental Psychology, University of Oxford, OX1 3UD Oxford, UK

## Abstract

Imagery plays an important role in our life. Motor imagery is the mental simulation of a motor act without overt motor output. Previous studies have documented the effect of motor imagery practice. However, its translational potential for patients as well as for athletes, musicians and other groups, depends largely on the transfer from mental practice to overt physical performance. We used bilateral transcranial direct current stimulation (tDCS) over sensorimotor areas to modulate neural lateralization patterns induced by unilateral mental motor imagery and the performance of a physical motor task. Twenty-six healthy older adults participated (mean age = 67.1 years) in a double-blind cross-over sham-controlled study. We found stimulation-related changes at the neural and behavioural level, which were polarity-dependent. Specifically, for the hand contralateral to the anode, electroencephalographic activity induced by motor imagery was more lateralized and motor performance improved. In contrast, for the hand contralateral to the cathode, hemispheric lateralization was reduced. The stimulation-related increase and decrease in neural lateralization were negatively related. Further, the degree of stimulation-related change in neural lateralization correlated with the stimulation-related change on behavioural level. These convergent neurophysiological and behavioural effects underline the potential of tDCS to improve mental and physical motor performance.

## Introduction

Motor imagery (MI) is defined as the mental simulation of a motor act without overt motor output^1^. Neural and behavioural evidence suggests similarities between the imagination and the actual execution of movements. For instance, studies using the mental chronometry paradigm, i.e. an experimental approach to measure cognitive processing speed, have demonstrated that the time needed to mentally and physically complete a movement is similar^2–4^. Moreover, MI follows the same physical laws as motor execution, like the speed-accuracy trade-off as stated in Fitt’s law^5,6^. Finally, changes in vegetative responses such as, for example, blood pressure and heart rate, that are associated with physical effort vary in a similar manner during movement imagination and execution^7^. Consistent with this, several neuroimaging studies comparing cortical and cerebellar activity during movement execution and imagination reported that MI engages almost the same networks as movement execution, albeit to a lesser extend^8–13^. Accordingly, both the execution and imagination of movements are associated with an event-related desynchronization (ERD) in the mu (8–12 Hz) and beta (13–30 Hz) frequency bands, which can be observed most prominently over sensorimotor cortical electrode sites in the electroencephalogram (EEG)^14^. This correspondence between the domains of movement execution and MI holds particular promise for stroke survivors as MI, and the associated activation of the sensorimotor cortex, can be exercised at all stages of recovery, even by the most severely paralysed patients^15^.

In addition to MI, transcranial direct current stimulation (tDCS) constitutes another promising approach to improve and restore motor functions. In the case of stroke, following the incident, experience-independent and experience-dependent changes on the neural and on the behavioural level can be beneficial, suboptimal, or even maladaptive^16^. Among the changes recognized as maladaptive is the widespread cortical activation of the unaffected hemisphere induced by attempted movements of the paretic upper limb^17–21^, which contrasts the more lateralized activity patterns seen in young healthy individuals. Thus, restoring the original contralateral dominance for unilateral upper limb movements in stroke survivors seems crucial. Previous studies used tDCS to modulate sensorimotor cortex activity either by directly stimulating the intact regions of the ipsilesional sensorimotor areas with anodal tDCS^22–26^ or indirectly through contralesional cathodal tDCS^22,26^. There is also evidence to suggest that the simultaneous application of ipsilesional anodal and contralesional cathodal tDCS may produce functional gains that are greater than either form of unilateral stimulation in isolation^27–32^ but see^33^. However, not much is known about how bilateral tDCS affects the neural correlates of MI or the relationship between stimulation-related change on neural and behavioural level.

Here, we evaluate the potential of MI combined with bilateral tDCS over sensorimotor cortices to modulate healthy older adults’ ERD and performance of a finger-tapping task. Healthy older adults constitute a good model for evaluating the merit of this approach, as it is well-established that the natural aging process is associated with a reduction in the hemispheric lateralization of cortical activation^34–38^, albeit typically to a lesser extent than in stroke survivors. Focussing on older adults avoids potential confounds that would be evident in a clinical study with stroke survivors (e.g. infarct location and type, lesion size, chronicity, medication). We hypothesized that the application of bilateral tDCS would increase the ERD lateralization for MI of the hand contralateral to the anode. We also hypothesized that a tDCS-related gain in neural lateralization would be associated with better performance on the motor task for the hand contralateral to the anode. Finally, the limited studies on this topic to date have primarily constrained the analyses to the hand contralateral to the anode^27,28,30,33,39^, we sought to validate and extend these findings by additionally evaluating how the hand contralateral to the cathode is affected and how the stimulation-induced effects are related.

## Results

### EEG data

**Figure 1:**
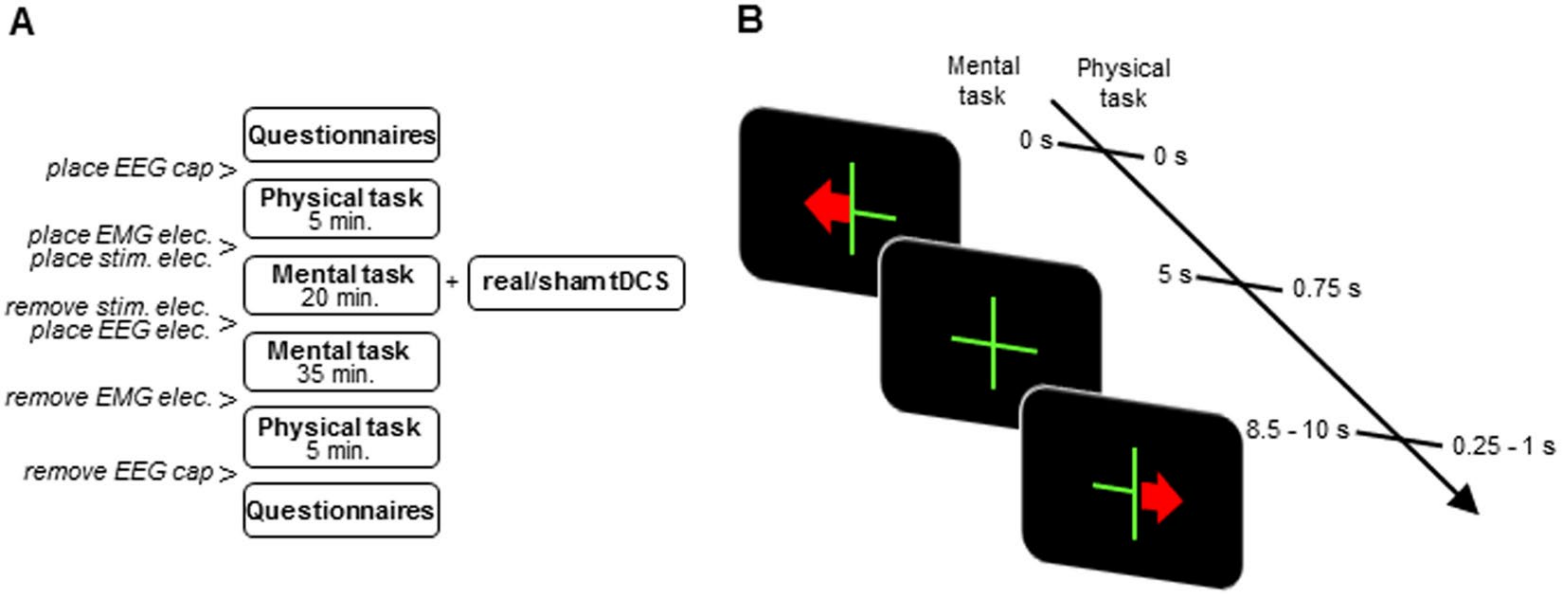
Schematic representation of the experimental procedure. (**A**) Illustrated is one of two sessions composing the within-subject sham-controlled design. Each session started with questionnaires followed by the physical motor task. After the physical motor task electrodes were placed over the *Opponens Pollicis* and *First Dorsal Interossei* of each hand to record surface EMG. Next stimulation was delivered, while participants performed the mental MI task. We administered either bilateral transcranial direct current stimulation (tDCS) or sham stimulation for 20 min. above the sensorimotor areas. After stimulation the stimulation electrodes were removed and replaced by EEG electrodes. Then the mental MI task was continued and MI-induced EEG activity was recorded. While participants performed the mental MI task, during and after stimulation, EMG activity was recorded. Following the MI task EMG electrodes were removed and the session ended with the physical motor task and questionnaires. (**B**) Trial layout for the mental MI task and the physical motor task. The presented stimuli (green fixation cross, red arrow pointing to the left or right) were identical for the mental MI and the physical motor task, while the timing of the stimulus presentation differed between the two tasks. The mental MI task followed the timing indicated on the left side of the time line and the physical motor task followed the timing indicated on the right side of the time line. During the physical motor task participants were instructed to place their index, middle, ring and little finger above predefined keys on the keyboard. Each trial started with the display of the red arrow pointing to the left or right, whereby the direction of the arrow indicated the hand to be used for the sequential finger-tapping task. Participants were instructed to initiate and execute the sequence as fast and accurate as possible. During the mental MI task participants were instructed to imagine the sequential finger-tapping task kinesthetically from the first person perspective for 5 s. Similar to the physical motor task each trial was initiated by the appearance of the red arrow and its direction indicated the hand to be used for the MI task.

**Figure 2:**
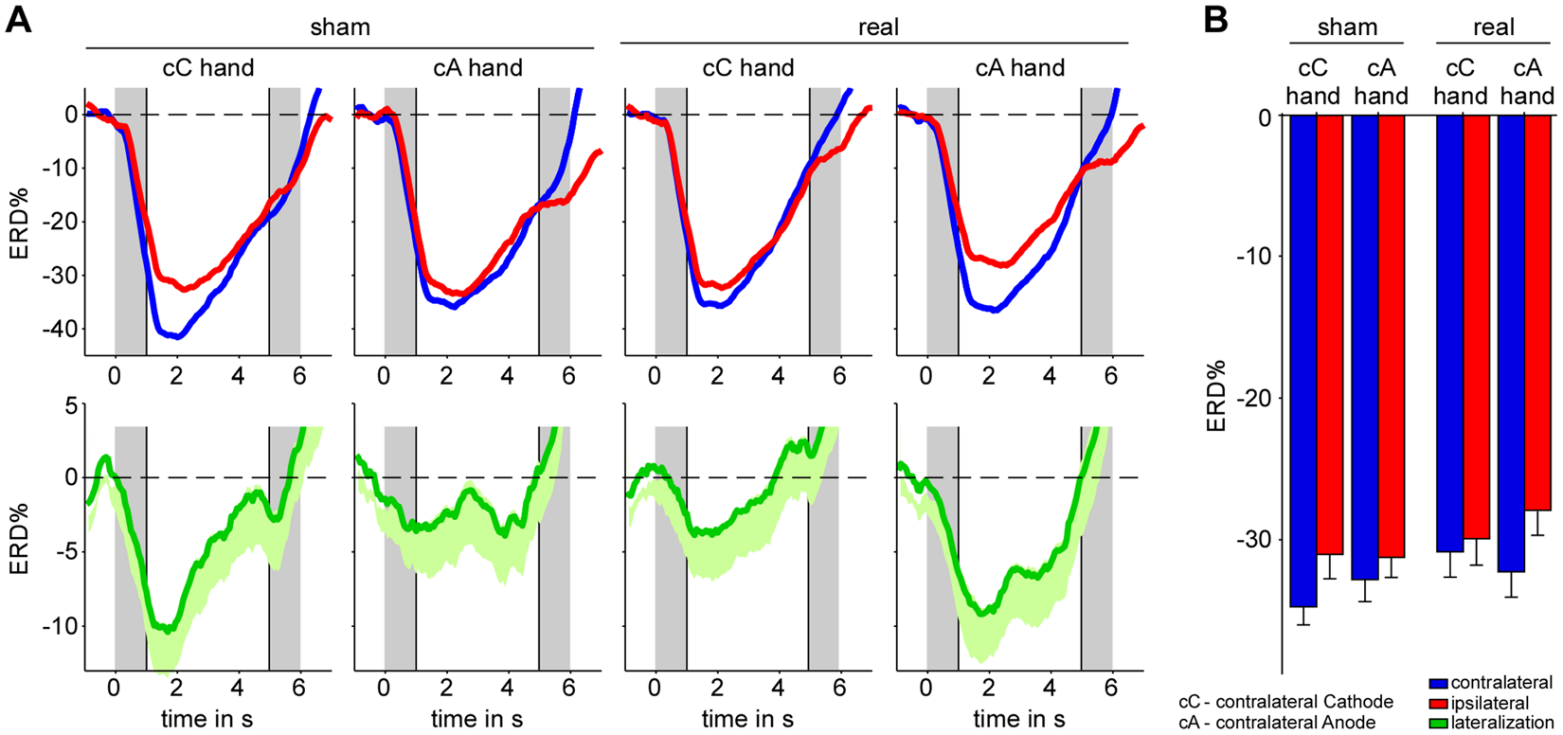
Descriptive statistics of stimulation-induced changes on neural level. (**A**) Grand average time course of MI-induced ERD% for the hand contralateral to the cathode (cC hand) and the hand contralateral to the anode (cA hand) separately for sham (left half) and real (right half) stimulation. For the sake of transparency contralateral ERD% and ipsilateral ERD% (top half) before calculating the ERD% lateralization (difference between contra- and ipsilateral ERD%, bottom half) used for the main analysis are also presented in this figure. Activity extracted above the contralateral and ipsilateral hemisphere, with respect to the hand used, is visualized in blue and red respectively. Activity was extracted from the scalp sites C3 and C4. The MI interval started at 0 s and ended at 5 s, however, as ERD% was derived using a moving average, the two grey shaded areas mark the time windows of begin and end of the MI interval. The two vertical lines indicate the time interval used for statistical analysis (0.5 s to 4.5 s) and the horizontal dashed line represents the zero line. For the ERD% lateralization the mean with standard error of the mean is illustrated. (**B**) MI-related activity extracted above the contralateral and ipsilateral hemisphere between 0.5 s to 4.5 s. Error bars represent one standard error.

**Figure 3:**
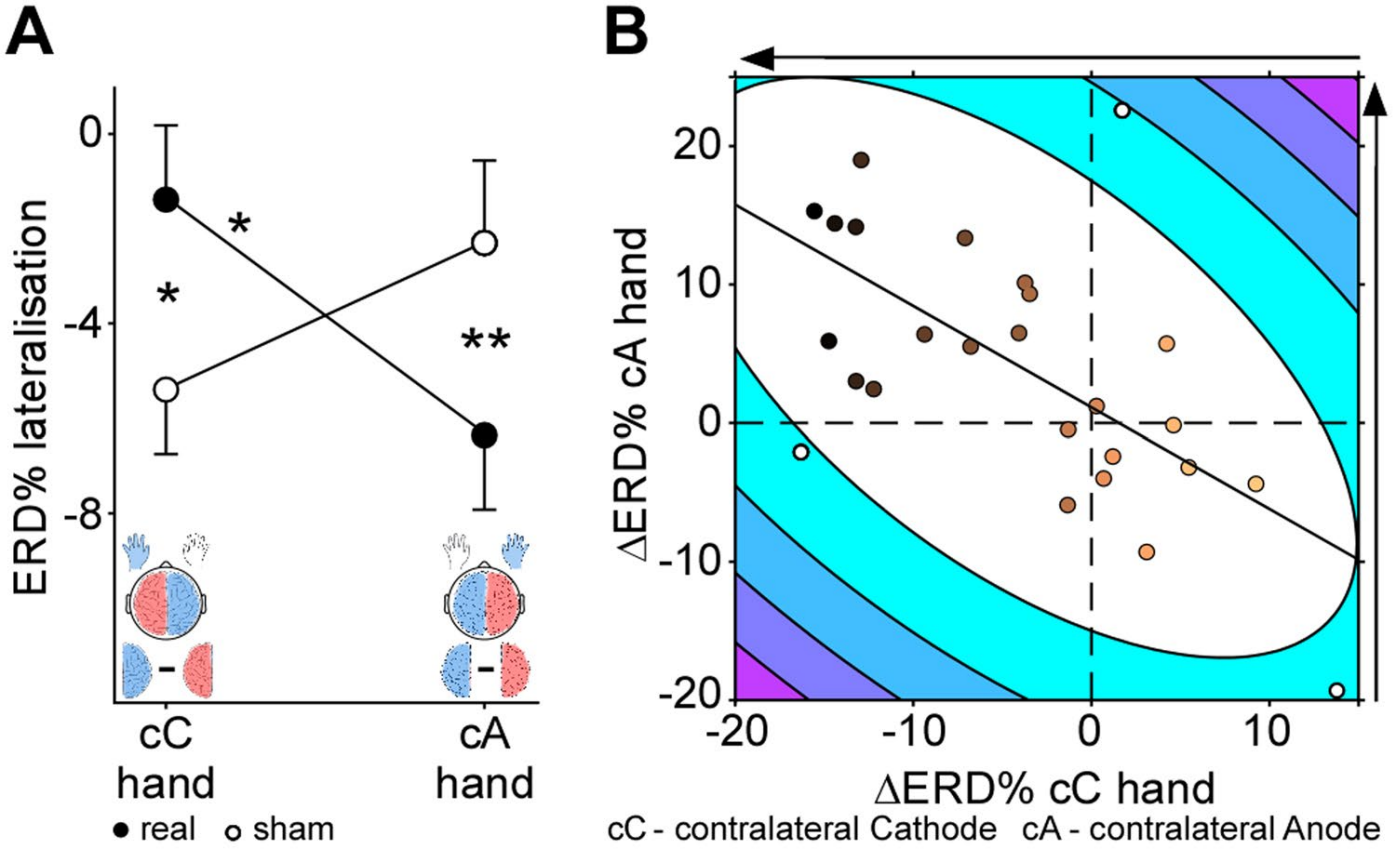
Interferential statistics of stimulation-induced changes on neural level. (**A**) Interaction between stimulation type and hand considering the degree of MI-induced ERD% lateralization. Error bars represent one standard error. *p < 0.05, **p < 0.01. (**B**) Association of MI-induced ΔERD% lateralization between both hands. Contour lines represent the bootstrapped Mahalanobis distance from the bivariate mean in steps of six squared units (purple colours indicate greater distance). White circles represent outliers. Darker colours of the other circles denote greater sensitivity to stimulation. The horizontal dashed lines mark zero and the solid line is the linear regression over the data after outlier removal. Direction of the arrows indicates the predicted stimulation-related change.

EEG data were recorded after 20 minutes of tDCS (see Fig. 1A). The ERD percentage change from baseline is denoted as ERD%, and the difference between sham and real stimulation is denoted as ΔERD%. Grand mean ERD% are summarized in Fig. 2. For the sake of simplicity we use the following terminology ‘hand contralateral to the cathode’ (abbreviated cC) and ‘hand contralateral to the anode’ (abbreviated as cA) to refer to the hand contralateral to the cathode and ipsilateral to the anode and to the hand contralateral to the anode and ipsilateral to the cathode, respectively. For both hands MI induced an ERD%, which was descriptively stronger, i.e. more negative, in the hemisphere contralateral to the implicated hand than in the ipsilateral hemisphere, irrespective of the stimulation condition, replicating previous findings^14^ (Supplementary Discussion). The effect of tDCS on the ERD% lateralization was compared using a 2 (stimulation type: sham/real) × 2 (hand: cC/cA) repeated measures analysis of covariance (ANCOVA) with stimulation order as a covariate. Central to the hypothesis, only the stimulation type x hand interaction reached significance (*F*_1,24_ = 11.52, *p* = 0.002, ${\eta }_{p}^{2}$ = 0.32, Fig. 3A), and no main effects were found for stimulation (*F*_1,24_ = 0.65, *p* = 0.427, ${\eta }_{p}^{2}$ = 0.03) or hand (*F*_1,24_ = 0.53, *p* = 0.475, ${\eta }_{p}^{2}$ = 0.02). Compared to sham stimulation, real stimulation was associated with stronger ERD% lateralization during MI of the hand contralateral to the anode (*F*_1,24_ = 10.82, *p* = 0.003, ${\eta }_{p}^{2}$ = 0.31; not corrected for multiple comparisons, two-tailed), and reduced ERD% lateralization during MI of the hand contralateral to the cathode (*F*_1,24_ = 7.10, *p* = 0.014, ${\eta }_{p}^{2}$ = 0.23). Notably, after real stimulation a significant difference in MI-related ERD% lateralization between the hand contralateral to the anode and the hand contralateral to the cathode was found (*F*_1,24_ = 7.12, *p* = 0.013, ${\eta }_{p}^{2}$ = 0.23), that was absent after sham stimulation (*F*_1,24_ = 1.95, *p* = 0.175, ${\eta }_{p}^{2}$ = 0.08). Importantly, the same analysis performed for the control sites, O1 and O2, did not result in any significant effects (all *p*s > 0.1) The relationship of MI-related ΔERD% lateralization between hands was examined using partial correlation analysis controlling for order of stimulation type. A significant negative relationship between MI-related ΔERD% lateralization for the hand contralateral to the anode and the hand contralateral to the cathode (*r*_*p*(23)_ = −0.58, *p* = 0.002) was observed, which remained after the removal of three outliers identified based on bootstrapping the Mahalanobis distance^40^ (*r*_*p*(20)_ = −0.71, *p* < 0.001; Fig. 3B).

### Behavioural data

**Figure 4:**
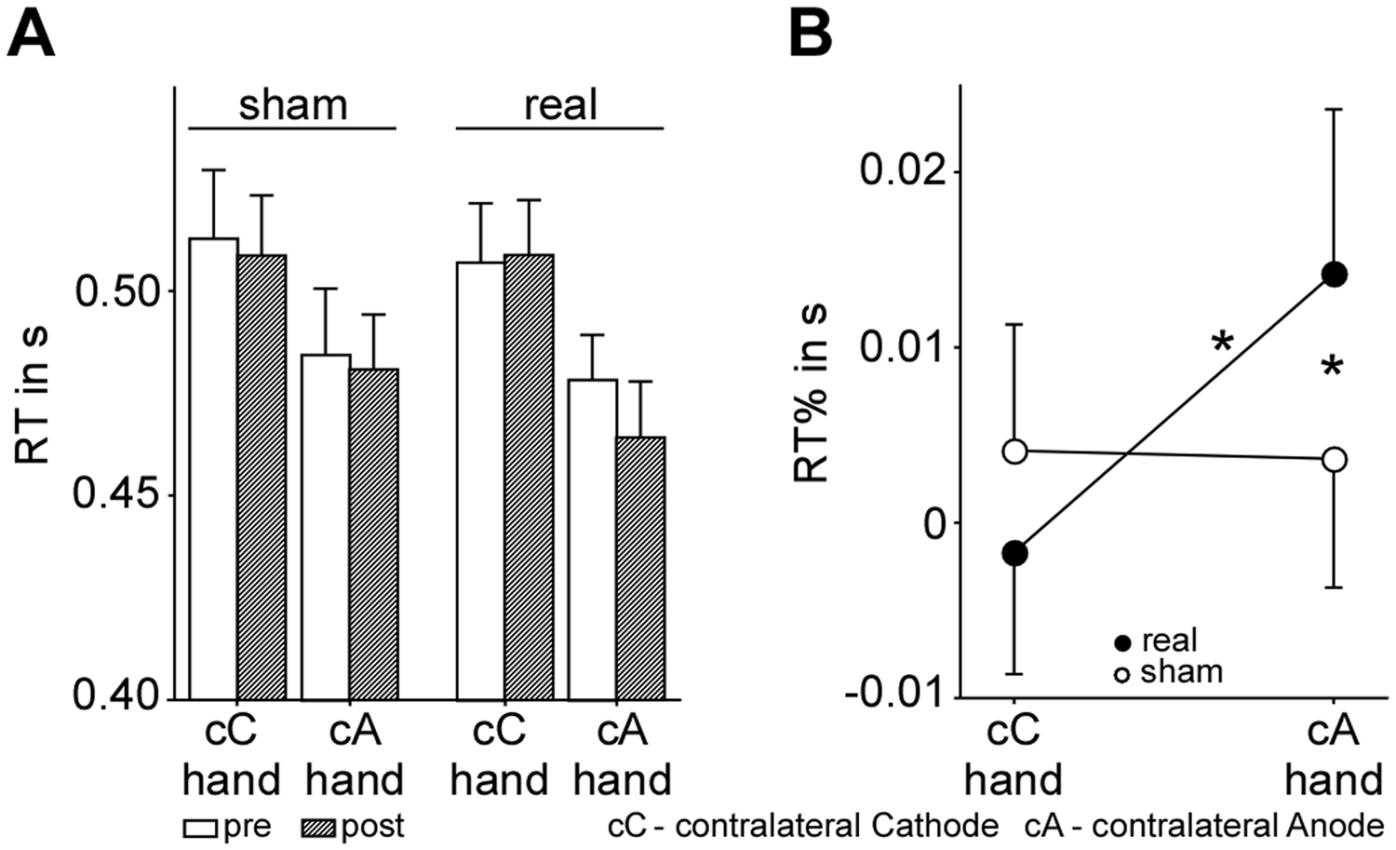
Descriptive and interferential statistics of stimulation-induced changes on behavioural level. (**A**) For the sake of transparency RTs of finger tapping before and after stimulation and subsequent MI practice before calculating the RT% (difference between RTs before and after stimulation) used for the main analysis is presented in this figure. Illustrated is the grand average RT for the hand contralateral to the cathode (cC hand) and the hand contralateral to the anode (cA hand) before (white) and after (grey) stimulation separately for sham and real stimulation conditions. Error bars represent one standard error. The anticipated difference between responses using the hand contralateral to the cathode (cC hand, left hand) and responses using the hand contralateral to the anode (cA hand, right hand) before stimulation is attributable to generally faster responses using the dominant right hand. (**B**) Interaction between stimulation type and hand considering the RT%. Error bars represent one standard error. *p < 0.05.

Behavioural data corroborate our neural findings. At the beginning and end of the experiment a physical sequential finger-tapping task was performed (see Fig. 1B). Note that the MI task was a covert task that did not yield any behavioural data. Grand mean reaction times (RTs) for the sequential finger-tapping task are summarized in Fig. 4A. The difference between RTs before and after stimulation is denoted as RT%, and the difference between sham and real stimulation is denoted as ΔRT%. RT% was compared using the same design used for the EEG analysis: a 2 (stimulation type: sham/real) × 2 (hand: cC/cA) repeated measures ANCOVA with stimulation order as a covariate. Similar to the EEG results, we observed a significant stimulation type x hand interaction (*F*_1,24_ = 5.07, *p* = 0.034, ${\eta }_{p}^{2}$ = 0.17; Fig. 4B), and no main effects for stimulation (*F*_1,24_ = 2.82, *p* = 0.106, ${\eta }_{p}^{2}$ = 0.11) or hand (*F*_1,24_ = 0.41, *p* = 0.530, ${\eta }_{p}^{2}$ = 0.02). In comparison to sham stimulation, real stimulation was associated with greater RT% (*F*_1,24_ = 7.08, *p* = 0.014, ${\eta }_{p}^{2}$ = 0.23; not corrected for multiple comparisons, two-tailed) for the hand contralateral to the anode, whereby the RT% for the hand contralateral to the cathode was not significantly decreased (*F*_1,24_ = 0.42, *p* = 0.522, ${\eta }_{p}^{2}$ = 0.02). The opposing polarity-depended effects resulted in a significant difference between RT% of the hand contralateral to the anode and RT% of the hand contralateral to the cathode (*F*_1,24_ = 4.70, *p* = 0.04, ${\eta }_{p}^{2}$ = 0.16), which was absent for the sham condition (*F*_1,24_ = 0.68, *p* = 0.417, ${\eta }_{p}^{2}$ = 0.03). In contrast to the neural level, no significant association between anodal-related increase and cathodal-related decrease in RT% was observed (*r*_*p(*23)_ = −0.20, *p* = 0.341; after removal of three outliers identified based on bootstrapping the Mahalanobis distance: *r*_*p*(20)_ = −0.14, *p* = 0.527, Supplementary Fig. 1).

Error rates for all conditions are illustrated in Supplementary Fig. 2. For statistical analysis of the error rates we employed the same factorial design as used for RT. The main effects of stimulation and hand, and the stimulation type x hand interaction, were not significant (all *p*s > 0.1).

### Relationship between EEG and behavioural data

**Figure 5:**
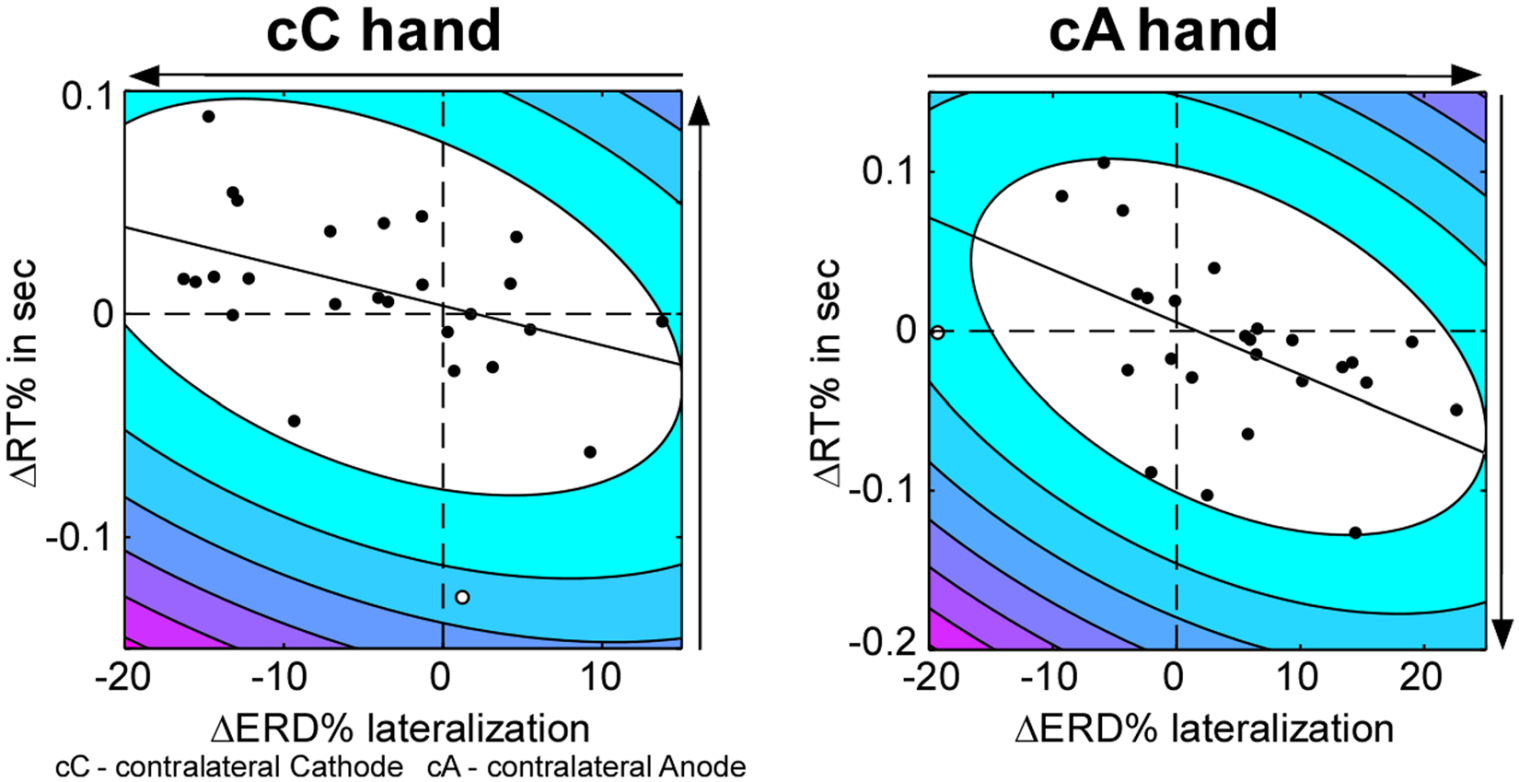
Association between ΔERD% lateralization and ΔRT% for the hand contralateral to the cathode (left half) and for the hand contralateral to the anode (right half). Contour lines represent the bootstrapped Mahalanobis distance from the bivariate mean in steps of six squared units (purple colours indicate greater distance). White circles represent outliers. The horizontal dashed lines mark zero and the solid line is the linear regression over the data after outlier removal. Direction of the arrows indicates the expected stimulation-related change.

To assess whether the observed changes on neural and behavioural level are related, we examined the relationship between tDCS-related changes in MI-induced ERD lateralization and RT difference separately for both hands using partial correlations controlling for order of stimulation. Significant negative associations between ΔERD% and ΔRT% were obtained for both, the hand contralateral to the cathode (*r*_*p*(23)_ = −0.45, *p* = 0.026; Fig. 5) and the hand contralateral to the anode (*r*_*p(*23)_ = −0.42, *p* = 0.036; Fig. 5). Notably, in both cases the relationship between the tDCS-induced changes in ERD% and RT% followed our prediction. When we removed the one outlier identified based on bootstrapping the Mahalanobis distance^40^, the respective correlations between ΔERD% and ΔRT% remained (*r*_*p(*22)_ = −0.51, *p* = 0.011; *r*_*p*(22)_ = −0.44, *p* = 0.033).

### Side effects and alternative explanations

To ensure that the observed differences between sham and real stimulation are not a consequence of differences in hand movements performed accidentally during MI (during or after stimulation), we analysed the electromyogram (EMG) data acquired during MI. EMG lateralization, as indicated by the difference between contra- and ipsilateral EMG activity, was analysed using two 2 (stimulation type: sham/real) × 2 (hand: cC/cA) repeated measures ANCOVA with stimulation order as a covariate. No significant main effects or interactions were observed on EMG lateralization either during or after stimulation (all *F*s < 1; *p*s > 0.1).

The level of discomfort reported by the participants was comparable during sham and real stimulation (*F*_1,24_ = 1.76, *p* = 0.198, ${\eta }_{p}^{2}$ = 0.07). Overall participants were not able to tell whether they received sham or real stimulation (*t*_1,25_ = 1.44, *p* = 0.16). Moreover, attention (*F*_1,24_ = 2.81, *p* = 0.107, ${\eta }_{p}^{2}$ = 0.11), motivation (*F*_1,24_ = 1.62, *p* = 0.216, ${\eta }_{p}^{2}$ = 0.06) and fatigue levels (*F*_1,24_ = 0.01, *p* = 0.923, ${\eta }_{p}^{2}$ < 0.01), which could have affected the pattern of results, were comparable between the two stimulation conditions. Finally, we confirmed that the intensity of MI was similar between real and sham stimulation (Supplementary Methods; left hand: *F*_1,24_ = 0.09, *p* = 0.772, ${\eta }_{p}^{2}$ < 0.01; right hand: *F*_1,24_ = 0.10, *p* = 0.756, ${\eta }_{p}^{2}$ < 0.01).

### Bayesian analysis

In addition to the frequentist statistics provided above, we also examined our main results according to bayesian statistics (see Supplementary Results).

## Discussion

We examined here how the application of bilateral tDCS over the sensorimotor cortices of older adults affects performance in a mental and a physical motor task. Consistent with our hypothesis, we observed a stimulation-dependent gain in MI-induced ERD lateralization and RT for the hand contralateral to the anode. In contrast, for the hand contralateral to the cathode, we observed that MI-induced ERD lateralization was reduced after real compared to sham stimulation but without accompanying behavioural change in RT. We furthermore observed that there was a significant correspondence between the gains in ERD lateralization for the hand contralateral to the anode, and the reduction in ERD lateralization for the hand contralateral to the cathode. Importantly, the respective gains and reductions in ERD lateralization were correlated with the changes in RT. These findings demonstrate the viability of bilateral tDCS as a tool for shaping the hemispheric lateralization patterns that are associated with MI and the performance in physical motor task.

MI for unilateral hand movements elicits an ERD stemming from sensorimotor areas^41^. Typically this ERD is more pronounced in the hemisphere contralateral to the implicated hand than in the ipsilateral hemisphere, leading to a specific lateralization pattern. However, following stroke, cortical lateralization, if seen at all, is typically very small in magnitude^17–20,42,43^. Moreover, a reduction in cortical lateralization is also related with normal aging^44^. Thus, the relatively modest degree of ERD lateralization we observed after sham stimulation is in line with previous fMRI^34–36,45–47^ and EEG^37,38,48–51^ studies investigating movement related activity in older adults.

Real tDCS stimulation led to significant changes in ERD lateralization. Specifically, relative to sham, real stimulation was associated with stronger MI-induced ERD lateralization for the hand contralateral to the anode. This finding is consistent with a previous study on MI and bilateral tDCS in stroke patients^39^, although Ang and colleagues focussed only on the degree of lateralization for the hand contralateral to the anode. However, the potential adverse effects of the bilateral montage on the hand contralateral to the cathode should not be neglected when considering the viability of this approach. Thus, we additionally examined the effect of stimulation on the hand contralateral to the cathode. Relative to sham, real stimulation was associated with a reduction in ERD lateralization induced by MI for the hand contralateral to the cathode. These opposing stimulation effects resulted in a significant difference in MI-induced ERD lateralization between both hands, which was absent after sham stimulation. Previous work has also shown that bilateral tDCS leads to polarity-dependent effects on motor evoked potentials^31^ but see^33^. We extend upon this here by demonstrating that there was a significant negative correlation between the amount by which ERD lateralization patterns were increased and decreased in response to real stimulation. The interindividual differences in the responsiveness to tDCS may depend on a wide range of variables (for a review on these aspects, see^52^). Advancing our understanding of these factors will have crucial implications for neurorehabilitation and cognitive enhancement. These findings clearly suggest that MI combined with tDCS is more effective at modulating hemispheric lateralization patterns than MI in isolation.

To further extend our knowledge about the relationship between mental practice and physical performance the effect of stimulation was investigated in a MI task and a physical motor task. As regards the physical motor task, compared to sham stimulation, real stimulation led to a significant improvement in RT for the hand contralateral to the anode. This finding is largely consistent with other studies examining the effect of bilateral tDCS on the hand contralateral to the anode showing RT modulation for the affected hand following stroke^27,28,30^ but see^33^. O’Shea and colleagues refer to differences in the stimulation protocol and hemispheric dominance as possible explanations for the divergent results^33^. Again, these studies focussed only on the hand contralateral to the anodal stimulation. Here, we did not find a significant change in physical performance for the hand contralateral to the cathode. However, real stimulation led to a significant RT difference between the hand contralateral to the cathode and the hand contralateral to the anode, and this effect was absent for sham stimulation. While on a neural level the opposing stimulation-related changes for the two hands were related, no such association was found on the behavioural level.

Importantly, stimulation-related modulation of MI-induced lateralization and stimulation-related modulation of RT difference was associated for both the hand contralateral to the cathode and the hand contralateral to the anode. These brain-behaviour correlations provide evidence for a common network underlying the mental and the physical task, as it has been argued before^53^.

Compared to sham, real stimulation led to significant changes during the mental task (ERD% lateralisation) and physical task (RT%) for the hand contralateral to the anode, whereas for the hand contralateral to the cathode significant changes were only observed during the mental task. The absence of a significant stimulation effect on performance of the physical task with the hand contralateral to the cathode may be at least partially attributable to the design of the study. For the sham condition, we observed that the RTs for both hands were slightly faster at the end of the session (post) compared to at the beginning (pre). A plausible explanation for this improvement may be a function of both increased practice of the physical task from pre to post measurement and the intermediary practice of a similar task through MI. Thus, for the hand contralateral to the anode, real stimulation likely resulted in an increase of the improvement seen in sham stimulation, whereas for the hand contralateral to the cathode, real stimulation may have counteracted the improvements seen in the sham stimulation. Consequently, a significant increase in RT would require a stronger inhibitory effect for cathodal stimulation than the facilitating effect required to see an improvement of the same size for anodal stimulation.

That said, previous work has advanced a number of other plausible explanations for why cathodal stimulation effects are less pronounced than anodal stimulation effects^22,54–57^ for review on these aspects, see^58,59^. For example it has been proposed that in the case of a high signal to noise ratio, anodal stimulation can increase the probability of firing and thus facilitate processing, while the same amount of cathodal stimulation is not sufficient to reduce the probability of firing and to hamper processing because the natural task-induced signal is still strong enough to elicit a response^59^. In the framework of motor rehabilitation the weaker effect of cathodal stimulation is advantageous, as bilateral tDCS does not necessarily involve a decline in motor performance for the unaffected hand.

We have demonstrated that a single session of bilateral tDCS induces polarity-dependent changes at the neural and the behavioural level, and that these measures correlate with one another. Future work should endeavour to identify the respective contribution of neural excitation and inhibition to the observed changes, and additionally establish the extent to which these findings generalise to left hemisphere stroke patients. This work should give particular consideration to how lesions in these patients may influence the current flow^60^. It will also be important to determine the persistence of the effects and how many sessions are needed to achieve long-lasting changes. Our findings impart novel insights on the viability of brain stimulation as a tool for modulating the hemispheric lateralization patterns associated with mental activity and the performance of a physical task, paving the way for future studies in stroke patients.

## Materials and Methods

### Participants

26 healthy older adults (13 females; mean age = 67.1 years; *SD* = 9.1 years) participated in this study, which was approved by the local ethics committee of the University of Oxford. All experiments were performed in accordance with relevant guidelines and regulations. Informed consent was obtained from all participants. All participants were free of neurological and psychiatric disorders, right-handed as indexed by the Edinburgh handedness inventory^61^, and had no prior experience with MI.

### Experimental design

The experimental procedure is illustrated in Fig. 1A. Throughout the session individuals were sitting in a comfortable chair. Each session started with questionnaires followed by a physical motor task. Next, stimulation was delivered, while participants performed the MI task, as the impact of brain stimulation depends among others on the state of the stimulated system^62^. After stimulation the MI task was continued and MI-induced activity was recorded using EEG. Subsequently the physical task was administered again. At the end participants were asked to rate the perceived intensity of the MI on a 5-point Likert scale. The scale constitutes the kinesthetic imagery subscale of the Kinesthetic and Visual Imagery Questionnaire (Supplementary Methods)^63^. Moreover, participants rated their level of discomfort, attention, fatigue and motivation on visual analogue scales.

The physical motor task lasted 5 min and consisted of 100 left and 100 right hand trials presented in random order. Stimulus presentation was controlled with OpenVibe Designer 1.0.1^64^. Participants were instructed to place their index, middle, ring and little finger above predefined keys on the keyboard. Each trial started with the display of a red arrow pointing to the left or right, see Fig. 1B. The direction of the arrow indicated the hand to be used for the sequential finger-tapping task (index – middle – ring – little finger), whereby each keystroke was recorded individually. Participants were instructed to initiate the sequence as fast and accurate as possible with the appearance of the arrow. After 0.75 s the arrow disappeared and in the inter-trial interval, lasting pseudorandomly for 0.25 to 1.0 s in steps of 0.25, a fixation cross was presented.

The MI task during and after stimulation was identical, with the sole exception of the number of trials performed. During stimulation two blocks comprising each 27 left and 27 right hand trials were conducted. The duration of each block was 8.3 min. After stimulation three blocks comprising each 33 left and 33 right hand trials were conducted. The duration of each block was 10.1 min. Between two consecutive blocks individuals rested for 2 min. Participants were instructed to sit as still as possible and to avoid eye-movements during this part of the experiment. Similar to the physical motor task each trial was initiated by the appearance of the red arrow and its direction indicated the hand to be used for the MI task, see Fig. 1. Participants were instructed to imagine the sequential finger tapping task (thumb to: index – middle – ring – little finger) kinaesthetically from the first person perspective^65^. After 5 s the MI interval was finished, which was illustrated by a replacement of the arrow by a fixation cross. The fixation cross remained the whole inter-trial interval lasting pseudorandomly for 3.5 s to 5.0 s in steps of 0.5 s. Please note that the physical motor task and the mental MI task followed different time scales. Neither for the physical nor for the mental motor task participants received any feedback about their performance.

### Transcranial direct current stimulation

Stimulation was delivered by a wireless DC brain stimulator (StarStim, NeuroElectrics, Barcelona, Spain), through a pair of 25 cm^2^ saline-soaked sponge electrodes. The current strength was 1.5 mA, resulting in a current density of 0.060 mA/cm^2^ at the surface of the scalp. The anode was placed over the left M1 (C3, according to the international 10/20-System) and the cathode over the right M1 (C4). Our motivation for using this electrode montage was based on the following lines of reasoning: (1) The effects of tDCS are known to be polarity-dependent such that cortical excitability is increased under the anode, and decreased under the cathode^66–68^; (2) While the present investigation involves healthy older adults, we envisage the value of extending the findings to research with stroke patients; (3) Following unilateral stroke, neural activity induced by movements of the affected limb is reduced in the affected, and increased in the unaffected, hemisphere; (4) The prevalence of left hemispheric stroke is much higher left hemispheric stroke^69^ and is also often associated with worse outcomes^69^. Hence, the electrode montage we employed here with the anode over the left M1 and the cathode over the right M1 would putatively be most appropriate for efforts to restore hemispheric lateralization following left hemispheric stroke.

The tDCS electrodes were encased in saline soaked sponges, and placed below the EEG cap in a manner that the metallic pins of the stimulation electrodes protruded through the placeholders of the EEG electrodes, which were for this part of the experiment removed from their placeholders. After the stimulation phase EEG electrodes replaced the stimulation electrodes without removing the cap. For the removal of the stimulation electrodes participants were asked to place both of their hands on electrode free positions of the cap to maintain its position. The examiner opened the velcro fastener and carefully removed the stimulation electrodes. After the removal of the stimulation electrodes the velcro fastener was closed again and the position of the cap verified by comparing the distances nasion – Cz – inion and left ear – Cz – right ear with measures obtained during initial EEG cap placement. EEG electrodes were inserted from the outside of the cap. Participants underwent both real and sham tDCS in a double-blind cross-over design. The double-blind design was realized by the double-blind mode of the control software (NeuroElectrics Instrument Controller v 1.4) and the Matlab interface for remote control v 2.3 (NeuroElectrics, Barcelona, Spain). Both conditions were separated by at least one week, with the order of the two conditions being randomized and counterbalanced. For the real stimulation condition the current was ramped up over 15 s, held constant for 20 min, and then ramped down over 15 s. For the sham condition, the same electrode montage was used, but after the current was ramped up over 15 s it ramped down and ceased after 15 sec. After completion of the two sessions participants were asked whether they could differentiate between the sessions with regard to stimulation condition^70^.

### Data acquisition

Both, EEG and EMG data were recorded with sintered Ag/AgCl electrodes and a 24-channel mobile EEG amplifier (mBrainTrain, Belgrade, Serbia). Data were recorded with a resolution of 24 bits and a sampling rate of 500 Hz and transmitted wirelessly via bluetooth to OpenVibe Acquisition Server 1.0.1^64^. The amplifier was tightly attached to a customized electrode cap (Easycap, Herrsching, Germany) specifically designed to record EEG and EMG data. More precisely, data were collected from twenty scalp sites (Fp1, Fp2, Fz, Fc1, Fc2, Fc5, Fc6, Cz, C3, C4, Cpz, Cp1, Cp2, Cp5, Cp6, Pz, P3, P4, O1, O2) and four EMG sites with Afz as ground and Fcz as reference. Surface EMG signals were recorded during MI (during and after stimulation) from both hands by placing electrodes over the *Opponens Pollicis* and *First Dorsal Interossei*. Concurrently to tDCS stimulation EEG data were recorded from a subset of electrodes (Fp1, Fp2, Fz, Cz, Cpz, Pz, O1, O2). Electrode-skin impedances were kept below 20 kΩ.

### EEG data analysis

EEG and EMG data were analysed using EEGLAB v12.0.2.6b^71^. EEG data were cleaned in a well-established two-step procedure. Firstly, stereotypical artefacts were corrected, secondly epochs containing non-sterotypical artefacts were rejected. Specifically, the attenuation of stereotypical artefacts was done with independent component analysis (ICA). To optimize ICA decomposition quality we performed several preprocessing steps before applying ICA. First, data were high-pass filtered (1 Hz, finite impulse response, filter order 1650) to remove low-frequency contributions (stationarity assumption). Second, data were low-pass filtered (40 Hz, finite impulse response, filter order 166) to remove high-frequency contributions (e.g. EMG activity, line noise) and subsequently data were down-sampled (250 Hz) to reduce computation time. Data containing non-sterotypical artefacts (e.g. gross head movements, swallowing) were removed. This was done by epoching the data into consecutive 1 s intervals and rejecting those containing non-typical artifact (EEGLAB functions pop_jointprob.m, pop_rejkurt.m, both SD = 3, rejected). For both tDCS sessions a comparable number of segments was excluded from a total of 1854 1 s segments (real: *M* = 417.0, *SD* = 107.6; sham: *M* = 418.0, *SD* = 105.8; *F*_*1,24*_ = 0.55, *p* = 0.464, ${\eta }_{p}^{2}$ = 0.023). This processing is considered an optimal trade-off for retaining much variance in the data but preventing the ICA algorithm being adversely affected by low- and high-frequency contributions as well as massive artefacts. Remaining data were submitted to extended infomax ICA^72^ to estimate the unmixing weights of 20 independent components. Components representing stereotypical artefacts such as eye blinks and eye movements were identified and removed from the raw data. The artefact corrected raw data were then high-pass filtered at 8 Hz (finite impulse response, filter order 825) and subsequently low-pass filtered at 30 Hz (finite impulse response, filter order 220). EEG data were segmented (−2 s to 7 s relative to the onset of the arrow). Segments containing residual movements identified by simultaneous EMG recordings were removed (see EMG Data Analysis). Additionally, any segments with residual artefacts were rejected (EEGLAB functions pop_jointprob.m, pop_rejkurt.m, both *SD* = 3). For both tDCS sessions a comparable number of trials was excluded (real: *M* = 28.2, *SD* = 10.1; sham: *M* = 29.2, *SD* = 7.1; *F*_*1,24*_ < 0.001, *p* = 1.000, ${\eta }_{p}^{2}$ < 0.001). ERD% was calculated as follows: ERD%(t) = (A(t) − R)/Rx100, where R is the power of a 1 s baseline interval (−2 s to −1 s before arrow onset), and A is the power at time point t, with t = 0 indicating the onset of MI^14^. For the statistical analysis of EEG data the ERD%, averaged across a time window covering 0.5 to 4.5 s with respect to the onset of the arrow, was extracted from the scalp sites C3, C4, O1 and O2, whereby the last two served as control sites.

### EMG data analysis

All EMG data, acquired during and after stimulation, were high-pass filtered at 10 Hz (finite impulse response, filter order 660) and cleaned from prominent electrical heartbeat activity by means of extended infomax ICA. Artefact-corrected EMG data were segmented into baseline intervals (−2.5 s to −0.5 s relative to the onset of the arrow) and MI intervals (0.5 s to 4.5 s with respect to the onset of the arrow). Two bipolar channels, one bipolar channel per hand, were formed and relative root mean square (RMS) values extracted. Relative RMS was defined as MI RMS minus baseline RMS. These RMS values were used for the analysis of EMG data acquired during stimulation. In contrast, EMG data acquired during MI after stimulation were analysed further. This further analysis aimed to identify and exclude segments containing minimal hand movements performed accidentally during MI to ensure that differences in actual movement between the two sessions do not bias the comparison between real and sham stimulation. To realize this, we implemented an automatic EMG analysis approach for identifying overt movements during MI. The same procedure was previously found to be effective for overt movement identification^38^. Trials containing minimal movements were identified by comparing the relative RMS values of the MI intervals for each hand and every trial to a threshold. The threshold for the detection of significant EMG activity was defined for each participant and stimulation condition individually as the average of the relative RMS plus/minus one standard deviation. Trials exceeding or falling below the threshold were labelled as trials containing residual movements and excluded from this EMG and EEG analysis. For both tDCS sessions a comparable number of trials was excluded from a total of 198 trials (real: *M* = 30.8, *SD* = 15.6; sham: *M* = 36.2, *SD* = 16.6; *F*_*1,24*_ = 2.43, *p* = 0.132, ${\eta }_{p}^{2}$ = 0.092).

### Behavioural data analysis

Behavioural data were analysed for left and right hand separately. Reaction time and percentage of correct trials were analysed for the first keystroke. RT means and *SDs* were calculated for the correct trials for each block and each participant after outlier removal (RTs > 1 s). On average RTs were calculated based on 86.52% (*SD* = 14.61%) of left hand trials and 86.67% (*SD* = 13.11%) of right hand trials. All statistical data analyses were performed using SPSS v23. All datasets generated during the current study are available from the corresponding author on reasonable request.

## Electronic supplementary material

Supplementary Information
