# Supplementary material for: Modulating hemispheric lateralization by brain stimulation yields gain in mental and physical activity
Source: Sci Rep. 2017 Oct 18;7:13430. doi: 10.1038/s41598-017-13795-1 (PMC5647441; doi:10.1038/s41598-017-13795-1)
Supplement: Supplementary file 1 — Supplementary Information [file 41598_2017_13795_MOESM1_ESM.pdf]

Supplementary Materials for "Modulating hemispheric lateralization by brain stimulation yields gain in mental and physical activity"

Catharina Zich, Siobhan Harty, Cornelia Kranczioch, Karen L. Mansfield, Francesco Sella, Stefan Debener, Roi Cohen Kadosh

Supplementary Methods

Intensity of motor imagery

At the end of each session participants were asked to rate the perceived intensity of the movement imagination on a 5-point Likert scale. The scale constitutes the kinesthetic imagery subscale of the Kinesthetic and Visual Imagery Questionnaire [1].

1. How intense was the sensation during left hand movement imagination?

5	4	3	2	1
As intense as executing the action	Intense	Moderately intense	Mildly intense	No sensation

2. How intense was the sensation during right hand movement imagination?

5	4	3	2	1
As intense as executing the action	Intense	Moderately intense	Mildly intense	No sensation

Supplementary Results

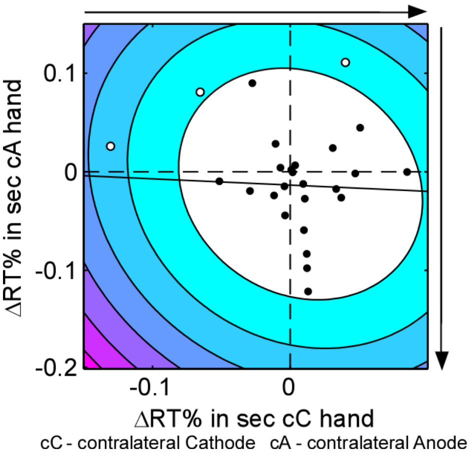

Supplementary Figure 1 Association of ΔRT% between both hands. Counter lines represent the bootstrapped Mahalanobis distance from the bivariate mean in steps of six squared units (purple colors indicate greater distance). White circles represent outliers. The solid line is the linear regression over the data after outlier removal. Direction of the arrows indicates the predicted stimulation-related change.

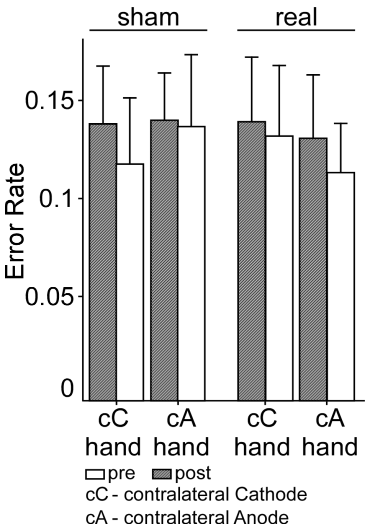

Supplementary Figure 2 For the sake of transparency error rates of finger tapping before and after stimulation and subsequent MI practice is presented in this figure. Grand average error rates for finger tapping performed with the hand contralateral to the cathode (cC hand) and the hand contralateral to the anode (cA hand) before (white) and after (grey) stimulation separately for sham and real stimulation conditions. Error bars represent one standard error. Errors comprise wrong responses and responses slower than 1 s.

Bayesian analysis

We tested the main results: the ANCOVA on ERD% lateralization, the ANCOVA on RT% and the three partial correlations according to Bayesian statistics. Statistical analyses were conducted using the free software JASP with default priors (JASP Team, 2017). We reported Bayes factors expressing the probability of the data given H1 relative to H0 (i.e., values larger than 1 are in favour of H1).

We run a Bayesian repeated measures ANCOVA on ERD% lateralization with Stimulation type (sham/real) and Hand (cC/cA) as within-subjects factors and Order as covariate (see Table S1). We specifically compared the model with main effects and interaction (Stimulation + Hand + Stimulation x Hand + Order) with the model with only the main effects (Stimulation + Hand + Order). The comparison yields a Bayes factor of 7.29 (i.e., 0.102/0.014) implying that the model including the interaction term is more in favour of H1 (moderate evidence) compared to the model without the interaction. It is important to note that while our aim was to specifically assess the interaction stimulation x hand, all the models provided weak evidence in favour of H1 (all $BFs_{10} < 1$).

Models	P(M)	P(M|data)	BF_M	BF_{10}	error %
Null model (incl. subject)	0.100	0.416	6.423	1.000	
Stimulation	0.100	0.084	0.823	0.201	0.731
Hand	0.100	0.107	1.081	0.257	3.717
Stimulation + Hand	0.100	0.020	0.186	0.049	1.645
Stimulation + Hand + Stimulation ∗ Hand	0.100	0.150	1.585	0.360	3.950
Order	0.100	0.120	1.225	0.288	0.591
Stimulation + Order	0.100	0.025	0.229	0.060	1.569
Hand + Order	0.100	0.030	0.276	0.071	1.787
Stimulation + Hand + Order	0.100	0.006	0.053	0.014	3.153
Stimulation + Hand + Stimulation ∗ Hand + Order	0.100	0.042	0.398	0.102	2.024

Note. All models include subject.

Table S1. Results from Bayesian analysis on ERD% lateralization with Stimulation type (sham/real) and Hand (cC/cA) as within-subjects factors and Order as covariate.

To further decompose the interaction between Hand and Stimulation we run a Bayesian repeated measures ANCOVA on ERD% lateralization only for real stimulation with Hand (cC/cA) as within-subjects factor and Order as covariate (see Table S2). The BF_{10} associated with Hand was 2.696 suggesting anecdotal evidence in favour of H1, that is, a difference between the two hands following real stimulation.

Models	P(M)	P(M|data)	BF_M	BF_{10}	error %
Null model (incl. subject)	0.250	0.204	0.767	1.000	
Hand	0.250	0.549	3.651	2.696	1.092
Order	0.250	0.066	0.214	0.327	0.515
Hand + Order	0.250	0.181	0.663	0.889	0.978

Note. All models include subject.

Table S2. Results from Bayesian analysis on ERD% lateralization for real stimulation with Hand (cC/cA) as within-subjects factor and Order as covariate.

Similarly, we run a Bayesian repeated measures ANCOVA on ERD% lateralization only for sham stimulation with Hand (cC/cA) as within-subjects factor and Order as covariate (see Table S3). The BF_{10} associated with Hand was 0.704 suggesting an anecdotal evidence in favour of H0, that is, no difference between the two hands following sham condition.

Models	P(M)	P(M|data)	BF_M	BF_{10}	error %
Null model (incl. subject)	0.250	0.427	2.235	1.000	
Hand	0.250	0.301	1.289	0.704	1.902
Order	0.250	0.162	0.581	0.380	0.461
Hand + Order	0.250	0.110	0.371	0.258	0.993

Note. All models include subject.

Table S3. Results from Bayesian analysis on ERD% lateralization for sham stimulation with Hand (cC/cA) as within-subjects factor and Order as covariate.

Moreover, we run a Bayesian repeated measures ANCOVA on ERD% lateralization only for cC hand with Stimulation (sham/real) as within-subjects factor and Order as covariate (see Table S4). The BF_{10} associated with Stimulation was 3.238 suggesting moderate evidence in favour of H1, that is, a difference between the two stimulation conditions for the cC hand.

Models	P(M)	P(M|data)	BF_M	BF_{10}	error %
Null model (incl. subject)	0.250	0.162	0.580	1.000	
Stimulation	0.250	0.525	3.310	3.238	1.350
Order	0.250	0.070	0.227	0.434	1.621
Stimulation + Order	0.250	0.243	0.964	1.501	1.636

Note. All models include subject.

Table S4. Results from Bayesian analysis on ERD% lateralization for cC hand with Stimulation (sham/real) as within-subjects factor and Order as covariate.

Finally, we run a Bayesian repeated measures ANCOVA on ERD% lateralization only for cA hand with Stimulation (sham/real) as within-subjects factor and Order as covariate (see Table S5). The BF_{10} associated with Stimulation was 1.884 suggesting anecdotal evidence in favour of H1, that is, a difference between the two stimulation conditions for the cA hand.

Models	P(M)	P(M|data)	BF_M	BF_{10}	error %
Null model (incl. subject)	0.250	0.250	1.000	1.000	
Stimulation	0.250	0.471	2.671	1.884	1.096
Order	0.250	0.096	0.318	0.384	1.487
Stimulation + Order	0.250	0.183	0.672	0.732	1.662

Note. All models include subject.

Table S5. Results from Bayesian analysis on ERD% lateralization for cA hand with Stimulation (sham/real) as within-subjects factor and Order as covariate.

We run a Bayesian repeated measures ANCOVA on RT% with Stimulation (sham/real) and Hand (cC/cA) as within-subjects factor and Order as covariate (see Table S6). We specifically compared the model with main effects and interaction (Stimulation + Hand + Stimulation x Hand + Order) with the model with only the main effects (Stimulation + Hand + Order). The comparison yields a Bayes factor of 0.5 (i.e., 0.014/0.028) implying that the model including the interaction term is more in favour of H0 (anecdotal evidence) compared to the model without interaction. However, all the models provide weak evidence in favour of H1 (all BFs_{10} < 1).

Models	P(M)	P(M|data)	BF_M	BF_{10}	error %
Null model (incl. subject)	0.100	0.437	6.986	1.000	
Stimulation	0.100	0.092	0.916	0.211	1.010
Hand	0.100	0.156	1.670	0.358	1.698
Stimulation + Hand	0.100	0.034	0.316	0.078	2.473
Stimulation + Hand + Stimulation ∗ Hand	0.100	0.017	0.153	0.038	2.102
Order	0.100	0.157	1.672	0.358	0.712
Stimulation + Order	0.100	0.033	0.309	0.076	1.514
Hand + Order	0.100	0.055	0.527	0.127	3.407
Stimulation + Hand + Order	0.100	0.012	0.112	0.028	7.057
Stimulation + Hand + Stimulation ∗ Hand + Order	0.100	0.006	0.055	0.014	6.068

Note. All models include subject.

Table S6. Results from Bayesian analysis on RT% with Stimulation type (sham/real) and Hand (cC/cA) as within-subjects factors and Order as covariate.

To further decompose the interaction between Hand and Stimulation we run a Bayesian repeated measure ANCOVA on RT% only for real stimulation with Hand (cC/cA) as within-subjects factor and Order as covariate (see Table S7). The BF_{10} associated with Hand was 0.905 suggesting anecdotal evidence in favour of H0, that is, no difference between the two hands following real stimulation.

Models	P(M)	P(M|data)	BF_M	BF_{10}	error %
Null model (incl. subject)	0.250	0.382	1.851	1.000	
Hand	0.250	0.345	1.582	0.905	1.307
Order	0.250	0.145	0.509	0.380	1.556
Hand + Order	0.250	0.128	0.441	0.336	1.747

Note. All models include subject.

Table S7. Results from Bayesian analysis on RT% for real stimulation with Hand (cC/cA) as within-subjects factor and Order as covariate.

Similarly, we run a Bayesian repeated measure ANCOVA on RT% only for sham stimulation with Hand (cC/cA) as within-subjects factor and Order as covariate (see Table S8). The BF_{10} associated with Hand was 0.280 suggesting moderate evidence in favour of H0, that is, no difference between the two hands following sham condition.

Models	P(M)	P(M|data)	BF_M	BF_{10}	error %
Null model (incl. subject)	0.250	0.409	2.075	1.000	
Hand	0.250	0.115	0.388	0.280	2.079
Order	0.250	0.371	1.768	0.907	0.450
Hand + Order	0.250	0.106	0.355	0.259	4.473

Note. All models include subject.

Table S8. Results from Bayesian analysis on RT% for sham stimulation with Hand (cC/cA) as within-subjects factor and Order as covariate.

Moreover, we run a Bayesian repeated measure ANCOVA on RT% only for cC hand with Stimulation (sham/real) as within-subjects factor and Order as covariate (see Table S9). The BF_{10} associated with Stimulation was 0.339 suggesting anecdotal evidence in favour of H0, that is, no difference between the two stimulation conditions for the cC hand.

Models	P(M)	P(M|data)	BF_M	BF_{10}	error %
Null model (incl. subject)	0.250	0.523	3.292	1.000	
Order	0.250	0.224	0.864	0.427	1.480
Stimulation	0.250	0.177	0.647	0.339	0.844
Order + Stimulation	0.250	0.076	0.246	0.145	1.852

Note. All models include subject.

Table S9. Results from Bayesian analysis on RT% for cC hand with Stimulation (sham/real) as within-subjects factor and Order as covariate.

Finally, we run a Bayesian repeated measure ANCOVA on RT% only for cA hand with Stimulation (sham/real) as within-subjects factor and Order as covariate (see Table S10). The BF_{10} associated with Stimulation was 0.443 suggesting anecdotal evidence in favour of H0, that is, no difference between the two stimulation conditions for the cA hand.

Models	P(M)	P(M|data)	BF_M	BF_{10}	error %
Null model (incl. subject)	0.250	0.496	2.958	1.000	
Order	0.250	0.193	0.715	0.388	1.699
Stimulation	0.250	0.220	0.847	0.443	1.236
Order + Stimulation	0.250	0.091	0.300	0.183	2.001

Note. All models include subject.

Table S10. Results from Bayesian analysis on RT% for cA hand with Stimulation (sham/real) as within-subjects factor and Order as covariate.

To account for the partial correlation analyses, we run a Bayesian linear regression analysis with ΔERD%_cA as dependent variable and ΔERD%_cC and Order as predictors (see Table S11). The model with ΔERD%_cC yields a BF_{10} of 20.285, thereby providing strong evidence in favour of a relation between ΔERD%_cA and ΔERD%_cC.

Model Comparison - Delta_ERD%_cA

Models	P(M)	P(M|data)	BF_M	BF_{10}	error %
Null model	0.250	0.033	0.102	1.000	
Delta_ERD%_cC	0.250	0.668	6.039	20.285	1.954e −4
Order	0.250	0.017	0.050	0.501	0.001
Delta_ERD%_cC + Order	0.250	0.282	1.181	8.576	4.384e −5

Table S11. Results from Bayesian linear regression analysis with ΔERD%_cA as dependent variable and ΔERD%_cC and Order as predictors.

We run a Bayesian linear regression analysis with ΔERD%_cA as dependent variable and ΔRT%_cA and Order as predictors (see Table S12). The model with ΔRT%_cA yields a BF_{10} of 3.085, thereby suggesting moderate evidence in favour of a relation between ΔERD%_cA and ΔRT%_cA.

Model Comparison - Delta_ERD%_cA ▼

Models	P(M)	P(M|data)	BF_M	BF_{10}	error %
Null model	0.250	0.171	0.619	1.000	
Order	0.250	0.086	0.281	0.501	0.001
Delta_RT%_cA	0.250	0.528	3.355	3.085	6.529e −4
Order + Delta_RT%_cA	0.250	0.215	0.823	1.257	9.618e −4

Table S12. Results from Bayesian linear regression analysis with ΔERD%_cA as dependent variable and ΔRT%_cA and Order as predictors.

We run a Bayesian linear regression analysis with ΔERD%_cC as dependent variable and ΔRT%_cC and Order as predictors (see Table S13). The model with ΔRT%_cC yields a BF_{10} of 2.450, thereby suggesting anecdotal evidence in favour of a relation between ΔERD%_cA and ΔRT%_cA.

Model Comparison - Delta_ERD%_cC

Models	P(M)	P(M|data)	BF_M	BF_{10}	error %
Null model	0.250	0.196	0.730	1.000	
Order	0.250	0.078	0.254	0.398	0.002
Delta_RT%_cC	0.250	0.480	2.766	2.450	7.086e −4
Order + Delta_RT%_cC	0.250	0.247	0.982	1.259	9.598e −4

Table S13. Results from Bayesian linear regression analysis with ΔERD%_cC as dependent variable and ΔRT%_cC and Order as predictors.

Supplementary Discussion

We noted that the degree of MI-induced ERD% lateralization was descriptively larger for the left hand (hand contralateral to the cathode) than for right hand (hand contralateral to the anode) following sham stimulation. A reanalysis of a previously published dataset [2] also revealed a descriptively, but not significantly, larger ERD% lateralization for left hand MI than for right hand MI in older adults. However, in this reanalysis younger adults exhibited the opposite pattern.

References

1. Malouin, F., Richards, C. L., Durand, A. & Doyon, J. Clinical assessment of motor imagery after stroke. *Neurorehabil. Neural Repair* **22,** 330–340 (2008).
2. Zich, C. *et al.* Lateralization patterns of covert but not overt movements change with age: An EEG neurofeedback study. *Neuroimage* **116,** 80–91 (2015).
